# Preservation of Anthocyanins in Postharvest Grapes Through Carboxymethyl Chitosan Films Containing Citrus Essential Oil Emulsion via Enzymatic Regulation

**DOI:** 10.3390/foods14122015

**Published:** 2025-06-06

**Authors:** Xinye Wu, Haiying Wang, Yuan Zhou, Wei Xi, Yiqin Zhang, Shanshan Li, Jiaying Tang, Suqing Li, Qing Zhang, Yaowen Liu, Jingming Li, Mingrui Chen, Wen Qin

**Affiliations:** 1 College of Food Science, Sichuan Agricultural University, Ya’an 625014, China; 2 Sichuan Advanced Agricultural & Industrial Institute, China Agricultural University, Chengdu 611430, China

**Keywords:** carboxymethyl chitosan, emulsion, grape, anthocyanin, enzyme activity

## Abstract

Carboxymethyl chitosan (CMCS) exhibits excellent film-forming capability but suffers from limited water resistance. To enhance hydrophobicity and antimicrobial properties, citrus essential oil was emulsified directly with citrus pectin and dispersed into the CMCS matrix. This study investigated the effects of varying emulsion concentrations (0, 1, 3, 5, and 7 wt%) on film performance. FT-IR, XRD, and SEM analyses confirmed uniform emulsion distribution within the CMCS matrix with favorable compatibility. Increased emulsion loading improved water resistance, antioxidant activity, and antimicrobial efficacy of the CMCS-based films, with the 3% emulsion concentration achieving optimal mechanical strength (TS: 4.09 MPa, EAB: 144.47%) and water vapor permeability (1.30 × 10^−10^ g·m·(Pa·s·m^2^)^−1^). Applied to grape preservation, the films significantly delayed quality deterioration of grapes. Furthermore, by modulating the activity of enzymes involved in anthocyanin metabolism, the films could effectively extend the shelf life of grapes by suppressing the oxidative degradation of anthocyanins.

## 1. Introduction

Grapes, as an important economic crop, contain various phenolic compounds—including bioactive substances such as anthocyanins, catechins, flavonols, and proanthocyanidins, which help to delay aging, alleviate fatigue, and prevent various cardiovascular diseases [1]. However, the contradiction between their high added value and their perishable nature has long constrained the sustainable development of the industry. The delicate texture, high water activity, vigorous respiration, and lack of a protective outer skin render the grapes particularly vulnerable to physiological, mechanical, and microbial damage after harvest [2]. In particular, the accumulation of anthocyanins imparts a red hue to the grape skin, affecting its external appearance. Anthocyanins gradually degrade during postharvest storage [3]. The metabolism of anthocyanins is mainly influenced by the synergistic action of several enzymes, such as phenylalanine ammonia-lyase (PAL), chalcone isomerase (CHI), chalcone synthase (CHS), flavanone-3-hydroxylase (F3H), and UDP-glucose flavonoid glycosyltransferase (UFGT), as well as by related degrading enzymes like peroxidase [4], polyphenol oxidase [5], and β-glucosidase (β-GC) [6,7,8].

Although traditional preservation techniques, such as low-temperature storage and chemical sanitizers, can temporarily inhibit postharvest decay and anthocyanin degradation, they face multiple challenges, including high energy consumption, the risk of chemical residues, and environmental unsustainability [9,10]. In recent years, active packaging based on natural biopolymers has attracted extensive research interest for fruit and vegetable preservation [11]. Carboxymethyl chitosan (CMCS) stands out due to its excellent biodegradability, film-forming capability, and antimicrobial activity. However, its strong hydrophilicity leads to inadequate barrier properties, limited mechanical strength, and lack of antioxidant functionality, severely restricting its application in high-moisture food preservation, particularly for fruits [12]. Current modification strategies face a “functionality-safety” trade-off dilemma: while physical blending with polysaccharides or proteins can improve mechanical properties, it struggles to simultaneously enhance hydrophobicity and antimicrobial efficacy; chemical cross-linking agents such as glutaraldehyde may strengthen the polymer network but introduce cytotoxic risks [13,14,15]. To address these challenges, constructing synergistic systems using natural components has emerged as a critical breakthrough.

Against this backdrop, citrus essential oil, containing active components like limonene and γ-terpinene, has been widely explored for its broad-spectrum antimicrobial and hydrophobic properties [16,17]. However, its high volatility and photosensitivity result in severe loss of active components [18]. Studies have demonstrated that emulsion encapsulation technology can significantly enhance essential oil stability. As a natural emulsifier, pectin exhibits good compatibility with CMCS. The acetyl groups and neutral sugar side chains in pectin anchor essential oil molecules through hydrophobic interactions, while carboxylic groups maintain emulsion dispersion homogeneity via electrostatic repulsion [19,20]. Integrating such emulsions into the CMCS matrix not only improves the hydrophobicity of films but also prolongs the antimicrobial activity cycle of essential oils through sustained-release mechanisms [21]. Therefore, incorporating pectin-stabilized essential oil emulsions is expected to enhance the mechanical properties of CMCS films while imparting additional antimicrobial and antioxidant functionalities. This composite design overcomes the limitations of CMCS and enhances preservation efficacy through synergistic effects of multiple bioactive components.

To date, research on pectin-based emulsion applications in films has primarily focused on composite emulsifiers [22]. Representative examples include citrus pectin and nanocrystalline cellulose-stabilized Pickering emulsion films [23], konjac glucomannan films enhanced by zein-pectin nanoparticle-stabilized oregano essential oil Pickering emulsions [24], and gelatin/sodium alginate films functionalized with persimmon pectin/ovalbumin-stabilized neem essential oil emulsions [25]. In contrast, studies investigating the modification of film properties using emulsions with pectin as the sole emulsifier remain relatively scarce. Moreover, there is limited research on how active films affect anthocyanin metabolism and related enzyme activities during the postharvest storage of grapes. In the present study, citrus pectin and citrus essential oil were used to prepare the emulsion. The effect of emulsion concentration on structures, mechanical properties, thermal stability, water resistance, optical properties, antioxidant, and antibacterial properties of CMCS films were studied. Additionally, the preservation efficacy of the films for grapes was evaluated, with particular emphasis on nutritional components and anthocyanin metabolism during postharvest storage of grapes. The results of this research will provide insights for the development of all-natural, plant-based, and multi-functional food packaging materials for potential application in fruit and vegetable preservation.

## 2. Materials and Methods

### 2.1. Materials

Grapes (*Vitis vinifera* “Muscat”) were purchased from a local farmers’ market (Ya’an, China). The citrus peel pectin (CP, degree of methyl esterification = 41.2%, total sugar content = 85.04%, arabinose/rhamnose/galactose/galacturonic acid = 20.7:4.1:14.4:45.8 mol%) and citrus essential oil (CEO, the major constituents comprised of D-limonene (75.071%), (+)-α-terpineol (8.863%), and γ-terpinene (4.677%)) were from our laboratory. Carboxymethyl chitosan (substitution degree ≥ 80%, CAS: 83512-85-0) was purchased from Ron Chemical Technology Co., Ltd. (Shanghai, China). *Staphylococcus aureus* (ATCC29213) and *Escherichia coli* (ATCC25922) were provided by the Key Laboratory Department of Agro-Products Processing and Storage, Sichuan Agricultural University (Ya’an, China).

### 2.2. Fabrication of Films

Firstly, CP solution (0.7 wt%) was mixed with CEO (5 wt%) and processed in a shear emulsifying machine (AngNing, Shanghai, China) at 10,000 rpm for 3 min, followed by ultrasonic treatment under an ice bath (400 W, 15 min, 2 s on/2 s off). Referencing the method established by Liu [26], the emulsion was added to CMCS solution (4 wt%) at concentrations of 1 wt%, 3 wt%, 5 wt%, and 7 wt%. In addition, glycerol (1 wt%) was added, and the total weight was controlled at 20 g. The mixture was homogenized at 10,000 rpm for 3 min. The degassed film-forming liquid was poured into 15 × 15 cm square dishes, dried at 40 °C for 12 h, and then equilibrated for 2 d at 25 °C, 60% RH. The prepared films were designated as EC0%, EC1%, EC3%, EC5%, and EC7%.

### 2.3. Characterization of Films

#### 2.3.1. Films Color and Light Transmission (LT)

The color of films was determined using a colorimeter (3NH, Zengcheng, China). The films were placed on a standard white board (L_0_ = 96.03, a_0_ = −0.28, b_0_ = 1.86) to determine the L, a, and b values, which were repeated five times for each sample. The total color difference (∆E) was calculated as Equation (1) [27]:

(1) $$\Delta E=\sqrt{{\left(L\;-\;L_{0}\right)}^{2}+{\left(\alpha \;-\;a_{0}\right)}^{2}+{\left(b\;-\;b_{0}\right)}^{2}}\;$$

where L, a, and b are the measured values of the films and L_0_, a_0_, and b_0_ are the initial values of the standard.

The LT of films was measured by UV–visible spectrophotometer (UV1901PC, Shanghai, China) over a scanning wavelength range of 200–800 nm^−1^.

#### 2.3.2. Mechanical Properties

The thickness of films was measured 10 times with a digital micrometer (Weidu Electronics, Wenzhou, China) at different locations [28]. The tensile strength (TS) and elongation at break (EB) of films were determined by a universal testing machine (Haida, Dongguan, China), and the experiments were repeated five times. The film was cut into 1 cm × 5 cm; the tensile rate was 20 mm·min^−1^, the scale length was 30 mm, and the trigger force was 50 N. TS and EB were calculated as Equations (2) and (3) [29]:

(2) $$\mathrm{TS}=\frac{F}{x\;\times \;W}$$

(3) $$\mathrm{EB}\left(\%\right)=\frac{\Delta L}{L_{0}}\;$$

where F, x, W, ΔL, and L_0_ are the tension (N), thickness (mm), width (mm), length increased at fracture (mm), and initial length of films (mm), respectively.

#### 2.3.3. Structural Characterization of Films

X-ray diffraction (XRD) data were measured by an X-ray powder diffractometer (Bruker D8 Advance, Berlin, Germany) with Cu Kα radiation (40 kV, 30 mA) with 2θ in the range of 5–45° at a rate of 5.0°·min^−1^. The peak area from XRD data was obtained after deconvolution of the spectra with the Peakfit^®^ 4.12 software (Systat software Inc., San Jose, CA, USA). Then, the degree of crystallinity of the films was calculated from the XRD data using the following formula [30]:

(4) $$\mathrm{Degree}\;\mathrm{of}\;\mathrm{crystallinity}\;\left(\%\right)=\left(1\;-\;\frac{A_{c}}{A_{c}+A_{a}}\right)\;\times \;100\%$$

where A_c_ and A*_a_* are the areas of the crystalline and amorphous phases, respectively.

Fourier transform infrared spectroscopy (FTIR) of films was collected using a Nicolet iS50 FTIR spectrometer (Thermo Fisher Scientific, Waltham, MA, USA) with an ATR sampling accessory, the resolution set to 4 cm^−1^, and a wavelength range from 4000 cm^−1^ to 720 cm^−1^. After films were embrittled using liquid nitrogen, their surfaces and cross-sections were observed with SEM (Hitachi Regulus SU8230, Tokyo, Japan) at an accelerating voltage of 3 kV.

#### 2.3.4. Differential Scanning Analysis (DSC)

Refer to the method by [31] with slight amendments. The melting point of films component was assessed by Q200DSC (TA, New Castle, DE, USA). Approximately 5 mg of films was added to an aluminum pan, the temperature range was 40–280 °C with a ramp rate of 10 °C·min^−1^.

#### 2.3.5. Water Vapor Permeability (WVP)

According to the method [32], the film was sealed to the top of a beaker containing anhydrous CaCl_2_ solution, then the beaker was placed in a desiccator containing a saturated NaCl solution (75% RH) and stored in an incubator at 25 °C. The change in weight of the cups was recorded at 12 h intervals for 3 days. WVP was calculated by Equation (5) [32]:

(5) $$\mathrm{WVP}=\frac{\Delta m\;\times \;H}{\Delta t\;\times \;A\;\times \;\Delta P}\;$$

where Δm, Δt, A, H, and ΔP are the weight difference (g), time difference (s), permeable area (m^2^), film thickness (m), and water vapor pressure difference between films.

#### 2.3.6. Water Contact Angle (WCA)

The WCA of films was analyzed using a contact angle meter (OCA20, Dataphysics, Filderstadt, Germany) according to the sessile drop method [33]. Three microliters of water was placed on the surface of films, and the WCA was measured after stabilizing for 30 s at 25 ± 1 °C.

#### 2.3.7. Antioxidant Activity

Based on methods [34], 100 mg of film sample was weighed and dissolved in 5 mL of distilled water. Subsequently, 1 mL of the film extract solution was mixed with 1 mL of DPPH solution (0.1 mM). A mixture of 1 mL of ultrapure water and 1 mL of DPPH solution (0.1 mM) was used as the control. The mixture was incubated at room temperature in the dark for 60 min. Then, the absorbance was measured at 517 nm by a Varioskan flash microplate reader (Thermo Fisher Scientific, Waltham, MA, USA). The DPPH radical scavenging activity of the film was calculated using Equation (6) [34]:

(6) $$\mathrm{DPPH}\;\mathrm{Radical}\;\mathrm{Scavenging}\;\mathrm{Activity}\;\left(\%\right)=\left(1\;-\;\frac{A_{1}}{A_{0}}\right)\;\times \;100\%\;$$

where A_0_ is the absorbance of the control, A_1_ is the absorbance of the sample solution.

#### 2.3.8. Antibacterial Activity

The antibacterial activity against *S. aureus* and *E. coli* was evaluated by the inhibition zone method [35]. The films were cut into discs (9 mm diameter), irradiated with a UV lamp for 30 min, and then placed on the surface of agar medium coated with the above pathogens (106 CFU mL^−1^) and incubated at 37 °C for 24 h. The diameter of the inhibition zone was measured by a digital caliper.

### 2.4. Preservation Experiment of Grapes

Grapes with uniform size, consistent maturity, and no mechanical damage were selected. Clusters were carefully detached with pedicels intact, washed with distilled water, air-dried, and placed in film-coated beakers (5 beakers per group, 20 grapes per beaker). Samples were stored at 15 °C and 75% RH throughout the storage of 18 d. Sampling occurred at intervals of 3 d for comprehensive parameter analysis. Fresh samples were immediately analyzed for titratable acidity (TA), total soluble solids (TSS), color difference, hardness, and weight loss rate. Besides, the other frozen sample was stored at −80 °C until remaining biochemical index analysis.

#### 2.4.1. Weight Loss Rate

The weight loss rate was determined by the weighing method. Five grapes were measured in each group, weighed and recorded at each sampling time. The weight loss rate was calculated by Equation (7) [36]:

(7) $$\mathrm{weight}\;\mathrm{loss}\;\mathrm{rate}\;\left(\%\right)=\frac{m_{0\;}-\;m_{t}}{m_{0}}\times 100\%\;$$

where m_0_ is initial weight of grapes (g), m_t_ is the weight of grapes on day t (g).

#### 2.4.2. Hardness

The hardness of grapes was determined using a fruit durometer (Edbao, Yueqing, China), the grapes were cut, and five points were randomly selected on the cut for measurement, and five grapes were randomly selected from each group. The results were averaged and expressed as N.

#### 2.4.3. Color of Grapes

Sample color was measured by colorimeter (3NH, China). Five grapes were selected from each group for color measurement, and the same parts of the grapes were determined each time.

#### 2.4.4. Total Soluble Solid (TSS) and Titratable Acidity (TA)

Ten grapes were randomly taken from each treatment group. They were juiced by a juicer (AUX, Ningbo, China), then centrifuged at 8000 rpm for 10 min, and the supernatant was collected. The TSS content was determined after zeroing the refractometer (lohand, Hangzhou, China) with distilled water. The TA content of grapes was determined by acid–base titration [2]. Three mL of the supernatant was taken and diluted to 45 mL. Two drops of 1% phenolphthalein were added to 10 mL of the diluted supernatant, and the solution was titrated through 0.1 mol·L^−1^ NaOH solution until the solution turned light pink and did not fade within 30 s. The consumed volume of NaOH solution was recorded, TA content was calculated using Equation (8) [37]:

(8) $$\mathrm{TA}\;\mathrm{content}\;\left(\%\right)=\frac{V\;\times \;C\;\times \;\left(V_{1}\;-\;V_{2}\right)\;\times \;K}{V_{0}\;\times \;m}\;\times \;100\%\;$$

where molar concentration of C is NaOH standard solution, mol/L, V is the total volume of sample filtrate, mL, V_0_ is the volume of filtrate taken during titration, mL, V_1_ is the volume of NaOH standard solution consumed during titration, mL, V_2_ is the volume of blank NaOH standard solution consumed, mL, K is the tartaric acid conversion coefficient, 0.075, and m is the sample mass, g.

#### 2.4.5. Total Phenolic Content (TPC) and Total Anthocyanins Content (TAC)

Grapes were ground to powder in liquid nitrogen, 1 g of sample was weighed, 20 mL of 70% precooled acidified methanol was added, ultrasonicated (480 W) at low temperature and in the dark for 30 min, centrifuged at 8000 rpm for 10 min, and the supernatant was collected for use.

TPC was determined by the Folin–Ciocalteu colorimetric method [38]. Briefly, 100 μL of supernatant and 50 μL of Folin phenol reagent (2 M) were added in turn, and then 150 μL of 20% Na_2_CO_3_ was added after 2 min. Finally, the mixture was made up with distilled water to a final volume of 1 mL, and incubated in the dark for 1 h. The absorbance value of the mixture was measured at 760 nm, TPC of grapes was calculated using gallic acid as the standard and expressed as milligrams of gallic acid equivalent per gram of grape (mg·g^−1^). Two grams of the frozen sample was taken and ground into powder in liquid nitrogen. The detection of anthocyanins was performed using a detection kit (Boxbio, Beijing, China) by the pH differential method. Briefly, 0.1 g of the sample was weighed and mixed with 1 mL of extraction buffer. The mixture was incubated at 60 °C for 30 min, then centrifuged at 12,000 rpm, 10 min at 4 °C. The supernatant was collected as the test sample. For analysis, 20 μL aliquots of the sample were separately added with Reagent 1 and Reagent 2, designated as A_test_ and B_test_, respectively. The absorbance values of A_test_ were measured at 530 nm and 700 nm, recorded as A_1_ and A_2_, while those of B_test_ at the same wavelengths were recorded as A_3_ and A_4_. Calculations were performed as follows: ΔA_test_ = A_1_ − A_2_, ΔB_test_ = A_3_ − A_4_, and ΔA = ΔA_test_ − ΔB_test_. TAC was calculated using Equation (9):

(9) $$\mathrm{TAC}\;(\mu \mathrm{mol}/g)=\frac{\Delta A\;\times \;V\;\times \;V_{a}\;\times \;10^{3}}{\varepsilon \;\times \;d_{1}\;\times {\;V}_{s}\;\times \;W}=\frac{0.743\;\times \;\Delta A}{W}$$

where V_s_ is the volume of the test sample added to the reaction system, 0.02 mL, V is the total reaction volume, 0.2 mL, V_a_ is the total extraction volume, 1 mL, ε is the molar extinction coefficient of anthocyanins (2.69 × 10^4^ mL·mmol^−1^·cm^−1^), d_1_ is the optical pathlength in the 96-well plate, 0.5 cm, and W is the sample mass, g.

#### 2.4.6. Enzyme Activities of Anthocyanin Metabolism

Firstly, 5 mL of PBS buffer (pH = 7.4) was added to 1 g of frozen powder sample, then homogenized and centrifuged at 4 °C (10 min, 10,000 rpm); the supernatant was used as enzyme extract. The activities of related enzymes (PAL, CHI, F3H, UFGT, and β-GC) were determined by the ELISA kits (Jiangsu Jingmei Biotechnology Co., Ltd., Yancheng, China) according to the manufacturer’s instructions.

### 2.5. Statistical Analyses

All experiments of grapes were repeated three times in parallel, and the results were expressed as the mean ± standard deviation. Statistical analyses were performed using IBM SPSS 26.0. The significance of differences in the means between groups was assessed using one-way analysis of variance followed by Duncan’s multiple range test. Differences were considered to be significant (*p* < 0.05). 

## 3. Results and Discussion

### 3.1. Characterization of Films

#### 3.1.1. Appearance and Light Transmittance of Films

Color serves as a critical quality indicator for films due to its direct impact on visual appearance. As shown in Table 1, increasing the concentration of citrus essential oil emulsion progressively reduced the L value (lightness) of CMCS films, while the a value (red-green degree) remained relatively stable except for a significant decrease at 7% loading content (*p* < 0.05). Concurrently, the b value (yellow-blue degree) exhibited a consistent upward trend, collectively indicating reduced brightness and a gradual shift toward a yellow-green hue in the CMCS film surface. This evolution could be attributed to the creamy-yellow to the emulsion system.

**Figure 1:**
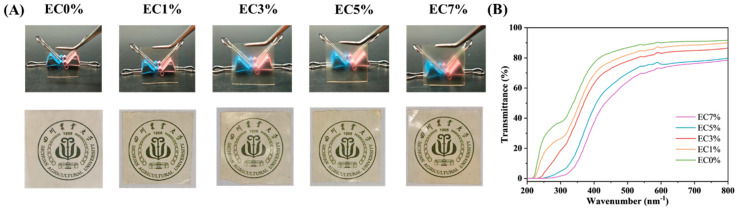
Appearance (**A**) and transmittance (**B**) of CMCS films containing 0%, 1.0% and 3.0%, 5.0%, 7.0% of citrus essential oil emulsion.

The light transmittance trend of CMCS films was consistent with its appearance and color changes. As shown in Figure 1A, higher citrus essential oil emulsion addition levels caused the films to become more yellow and opaque. This color change can reduce visible light and UV exposure, thereby preventing oxidative spoilage and quality degradation in packaged foods. Within the 200–800 nm^−1^ range, EC0% (pure CMCS film) exhibited the highest transmittance and appeared transparent. After citrus essential oil emulsion addition, the dispersed droplets in the matrix solution caused observable changes. Compared to the control film, samples starting from EC3% showed a distinct frosted texture. The transmittance of emulsion-containing films decreased progressively with increasing citrus essential oil emulsion content. The transmittance in the 200–400 nm revealed that all emulsion-containing films exhibited enhanced UV resistance regardless of loading levels, particularly EC7%. It blocked most UVB (275–320 nm^−1^) and nearly all UVC (200–275 nm^−1^) radiation (Figure 1B). This phenomenon may be attributed to the ultraviolet-absorbing capacity of aromatic groups present in the essential oil [39]. In line with the present finding, compared to pure collagen films, cinnamon essential oil emulsion-incorporated membranes demonstrated enhanced blocking abilities against both visible and ultraviolet light, particularly within the 250–320 nm UV range [40]. This UV-blocking property proves advantageous for food packaging applications, as ultraviolet radiation induces lipid oxidation, vitamin degradation, and off-flavor formation [41].

#### 3.1.2. Structural Characterization of Films

**Figure 2:**
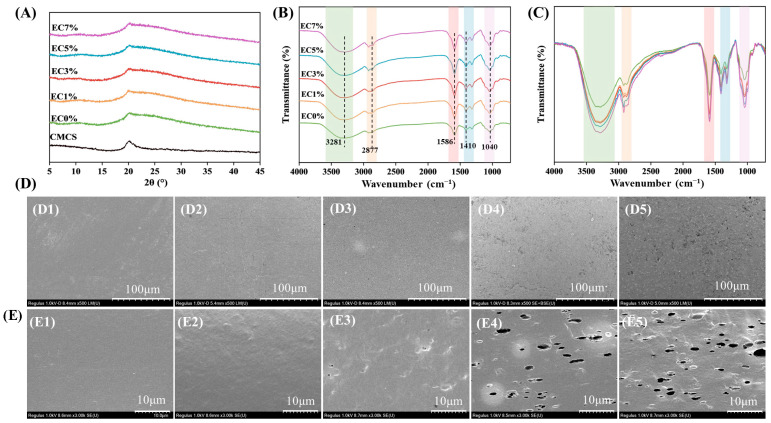
XRD (**A**), FT-IR (**B**), DSC (**C**), surface morphology (**D1**–**D5**, 500×) and cross-section morphology (**E1**–**E5**, 3.00k×) of CMCS films containing 0%, 1.0% and 3.0%, 5.0%, 7.0% of citrus essential oil emulsion.

X-ray diffraction (XRD) analysis was employed to characterize the crystalline structures in films. All groups (EC0%, EC1%, EC3%, EC5%, EC7%) exhibited a peak at approximately 20.1°, which corresponds to the characteristic diffraction peak of CMCS (Figure 2A). Notably, compared to pristine CMCS powder, the EC0% and other films exhibited broader and weaker diffraction peaks. Additionally, the crystallinities of the films (EC0%, EC1%, EC3%, EC5%, and EC7%) were determined to be 16.78%, 14.59%, 14.23%, 13.88%, 11.05%, and 11.23%, respectively, indicating that the addition of emulsion slightly reduced the crystallinity of the films. It likely due to the addition of glycerol and emulsion as plasticizers disrupting CMCS intermolecular hydrogen bonds and crystalline regions. This result indicated that CMCS exhibits good compatibility with the citrus essential oil emulsion in the composite films.

Fourier-transform infrared (FTIR) spectroscopy was employed to investigate structural interactions within the CMCS films. The EC0% film exhibited a broad absorption band spanning 3700–3000 cm^−1^ (Figure 2B,C), corresponding to overlapping N-H and O-H stretching vibrations, with additional contributions from intermolecular hydrogen bonding [42]. Characteristic peaks at 1586 cm^−1^ and 1410 cm^−1^ were assigned to asymmetric and symmetric stretching modes of carboxylate anions, respectively [43]. Critically, no new absorption bands emerged in emulsion-containing films, confirming that interactions between CMCS and citrus essential oil emulsion were strictly physical in nature (van der Waals forces and hydrogen bonding), without covalent bond formation [44]. In addition, Figure 2C demonstrates a significant increase in the peak intensity of characteristic bands upon emulsion addition, indicating enhanced hydrogen bonding interactions between carboxylate functional groups of CMCS and the emulsion matrix. A similar shift in spectral peaks was previously observed by Yang et al. in *Litsea cubeba* oil–gelatin composite films [45]. The surface and cross-sectional morphologies of films were characterized by SEM analysis, with micrographs of films containing varying emulsion concentrations presented in Figure 2D. SEM analysis revealed that the EC0% film showed a uniformly dense and smooth texture in both surface and cross-sectional views, with no visible bubbles or wrinkles, confirming the excellent film-forming capability of CMCS. When citrus essential oil emulsion emulsions were added, droplets distributed evenly within the film matrix, disrupting the continuous structure of pure CMCS films. While EC1% appeared nearly identical to EC0%, structural changes became evident at 3% emulsion loading, where minor wrinkles emerged in cross-sections. At 5% loading, irregular pores of varying sizes formed in cross-sections, and surfaces became noticeably rougher. As depicted in Figure 2E5, the EC7% film exhibited significantly increased pore density across both surface and cross-sectional areas, suggesting excessive emulsion loading compromised the physical integrity of the film. The surface roughness observed in emulsion-containing films likely stemmed from droplet migration toward the air–film interface during drying, while pore formation may have resulted from water evaporation around coalesced oil droplets [46]. These morphological characteristics demonstrated congruence with prior observations in marjoram essential oil emulsion pectin-based composite films, which exhibited analogous surface texturing profiles and hierarchical porosity [47]. Collectively, these findings highlight the importance of optimal emulsion loading for maintaining dense and uniform microstructures.

#### 3.1.3. Mechanical Properties

**Figure 3:**
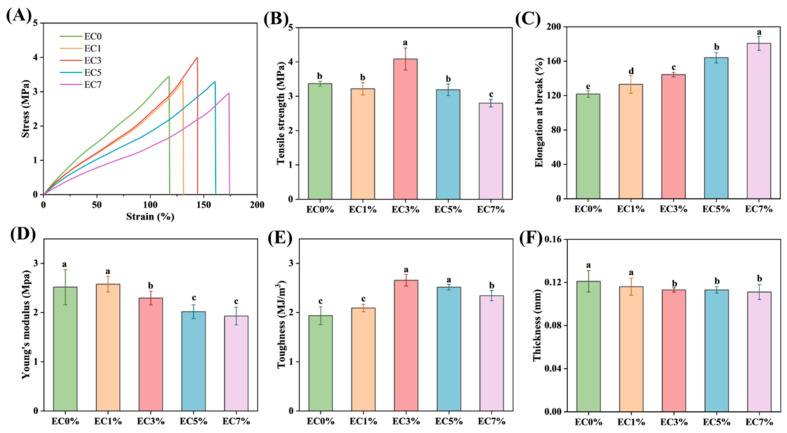
Stress–strain curves (**A**), tensile strength (**B**), elongation at break (**C**), young’s modulus (**D**), toughness (**E**) and thickness (**F**) of CMCS films containing 0%, 1.0% and 3.0%, 5.0%, 7.0% of citrus essential oil emulsion. Different minuscule letters indicate significant differences between groups (*p* < 0.05).

Tensile strength (TS) and elongation at break (EAB) exhibit a direct correlation with the chemical architecture of edible films, serving as the primary metrics for evaluating their mechanical performance [48]. When the citrus essential oil emulsion content increased from 0% to 3%, the TS of CMCS films showed an upward trend (Figure 3B). However, further increasing the emulsion content to 5% resulted in decreased TS, while the EAB continued to improve. Scanning electron microscopy images revealed that CMCS films with up to 3% emulsion content exhibited a dense microstructure without pores or cracks (Figure 3C). This emulsion integration enhanced plasticization effects: amphiphilic molecules in the emulsion embedded between CMCS chains through hydrophobic interactions, improving molecular chain slippage; simultaneously, surfactants in the emulsion formed hydrogen-bonding networks with polysaccharide hydroxyl groups, strengthening three-dimensional cross-linking density. These combined effects improved both TS and EAB (Figure 3C) [49,50]. These findings align with observations in konjac glucomannan composite films containing oregano essential oil emulsions [24]. When citrus oil emulsion loading exceeded 5%, excessive self-aggregation caused non-uniform stress distribution and increased pore density with enlarged cross-sectional dimensions (Figure 2E4,E5), ultimately reducing TS. Young’s modulus (YM) reflects film stiffness, with stiffer materials showing higher YM. Low citrus essential oil emulsion concentrations (≤3%) had no significant impact on YM (*p* < 0.05), but higher loadings decreased YM of the CMCS films, reducing stiffness while increasing toughness (Figure 3D,E). Previous studies have reported decreased TS in konjac glucomannan films upon direct addition of basil essential oil. Conversely, incorporation of Pickering emulsion-encapsulated essential oil significantly enhanced both TS and EAB, demonstrating that emulsion-based systems improve mechanical properties [44]. The CMCS film thickness exhibited negligible variation under controlled fabrication processes. However, a slight thickness reduction was observed with increasing citrus essential oil emulsion loading, attributed to decreased mass proportion of CMCS solids in the film-forming solution (Figure 3F).

#### 3.1.4. Thermal Property

**Figure 4:**
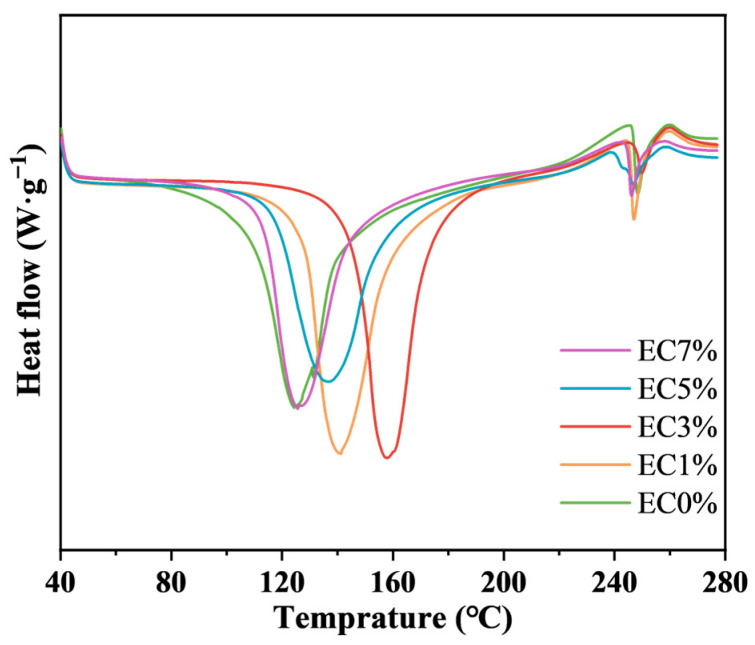
DSC of CMCS films containing 0%, 1.0% and 3.0%, 5.0%, 7.0% of citrus essential oil emulsion.

The effect of emulsion incorporation on the thermal properties of the CMCS films were investigated via differential scanning calorimetry (DSC). As shown in Figure 4, the primary endothermic peaks of all films occurred below 180 °C, attributed to overlapping water evaporation and essential oil volatilization. A secondary peak observed around 250 °C corresponded to structural decomposition of CMCS [51]. Compared to EC0%, citrus essential oil emulsion-modified films exhibited higher oil melting and water evaporation temperature ranges. The glass transition temperature [52] increased from 125 °C to 158 °C as emulsion loading rose to 3 wt%, indicating enhanced thermal stability due to oil–polymer interactions. However, excessive emulsion (>3 wt%) destabilized the film network through surface oil accumulation, leading to reduced thermal stability [53].

#### 3.1.5. Water Barrier Properties

**Figure 5:**
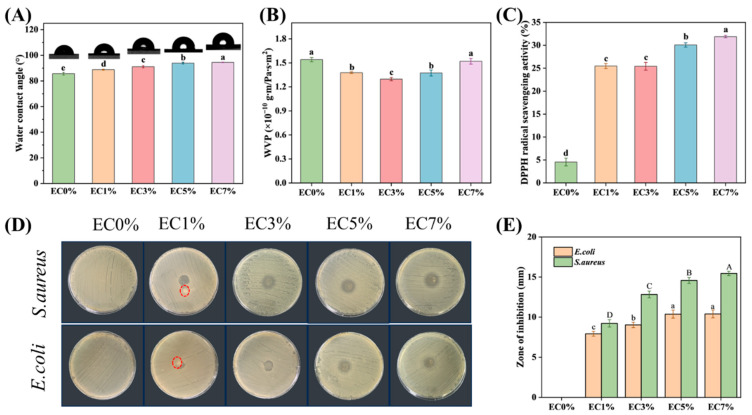
WVA (**A**), WVP (**B**), DPPH radical scavenging activity (**C**), zone of inhibition of CMCS films containing 0%, 1.0% and 3.0%, 5.0%, 7.0% of citrus essential oil emulsion (**D**,**E**). The red circle indicates the dissolved films. Different letters indicate significant differences (*p* < 0.05).

Surface hydrophobicity was determined by water contact angle (WCA). The WCA value θ > 90° has been identified as low wettability [54]. Pure EC0% showed low hydrophobicity, while the presence of hydrophobic dispersed phases, even at low levels, restricted water vapor transfer by interfering with hydrophilic domains and increasing the mass transfer tortuosity factor [55]. As shown in Figure 5A, the WCA of CMCS films increased from 85.63° to 94.53° with an increase in citrus essential oil emulsion loading (0–7 wt%), which may be attributed to reduced exposure of hydrophilic groups by emulsion-encapsulated essential oil. Additionally, enhanced hydrophobicity correlates with surface roughness, where rougher structures intensify this property [56]. Therefore, the rough surface observed in CMCS films with high concentrations of citrus essential oil emulsion (5 wt% and 7 wt%) also contributed to the higher WCA. Water vapor permeance (WVP) critically impacts the preservation efficacy of food packaging films. As depicted in Figure 5B, emulsion-incorporated films exhibited reduced WVP compared to EC0% films, demonstrating enhanced hydrophobicity. Among all samples, EC3% achieved the lowest WVP (17.5% reduction versus EC0%). However, WVP increased to 1.52 × 10^−10^ g·m·(Pa·s·m^2^)^−1^ at 7% citrus essential oil emulsion loading. Notably, while EC7% exhibited higher WCA than EC5%, its WVP increased correspondingly. This divergence may stem from stronger dependence of WCA on surface hydrophobicity, whereas WVP is predominantly governed by internal film architecture. As evidenced by SEM observations, EC7% displayed increased porosity, potentially accounting for its elevated WVP [40].

#### 3.1.6. Antioxidant, Antibacterial Effects of Films

The antioxidant activity of emulsion-incorporated and pure CMCS films was evaluated via DPPH radical scavenging. As illustrated in Figure 5C, EC0% films demonstrated minimal radical scavenging capacity (4.55 ± 0.85%), indicating negligible antioxidant potential. Emulsion-incorporated films exhibited significantly enhanced DPPH scavenging activity compared to EC0% (*p* < 0.05), with progressive improvements observed with increasing citrus essential oil emulsion loading. EC7% achieved the highest scavenging rate of 31.89 ± 0.27%. The primary chemical constituents in CEO (limonene, citral, and pinene) exhibit antioxidant capacity [57]. Their hydroxyl groups enable hydrogen transfer to stabilize free radicals, terminating chain reactions [49,58], thereby enhancing antioxidant properties of CMCS films. The observed trend in antioxidant activity aligns with findings reported by Liu [16], demonstrating gradual enhancement of CMCS/peach gum polysaccharide film antioxidant capacity with increasing concentrations of citrus essential oil emulsion.

The antimicrobial efficacy of CMCS films was assessed via the inhibition zone method. As shown in Figure 5D,E, both EC0% and EC1% films exhibited partial dissolution (red-circled areas). After 24 h incubation, EC0% films completely dissolved into the medium without forming inhibition zones, demonstrating their poor water resistance and lack of antimicrobial activity against both pathogenic strains (Figure 5D). Increasing CEO emulsion loading enlarged inhibition zone diameters due to elevated essential oil content. Monoterpenes (D-limonene) and phenolic compounds in citrus essential oil exerted antimicrobial effects by adhering to bacterial surfaces, targeting membrane-associated enzymes or plasma membranes. This mechanism compromised cell wall integrity, increased membrane permeability, and induced cytoplasmic leakage, ultimately achieving antibacterial effects [59]. Notably, at equivalent emulsion concentrations, the films demonstrated superior inhibitory effects against Gram-positive bacteria compared to Gram-negative strains. EC7% exhibited inhibition zone diameters of 10.40 ± 0.48 mm (*E. coli*) and 15.45 ± 0.27 mm (*S. aureus*) (Figure 5E). This disparity may originate from the additional outer membrane in Gram-negative bacteria, where the lipopolysaccharide layer impedes diffusion of hydrophobic compounds [60,61]. This phenomenon is consistent with the findings of Tanzeela [62], who observed that increasing essential oil concentration in citrus pectin films progressively enhanced antioxidant activity and strengthened antibacterial efficacy against *S. aureus*.

### 3.2. Preservation Effects of CMCS Films Loaded CEO Emulsion Films on Grapes

#### 3.2.1. Changes in Appearance and Color of Grapes

**Figure 6:**
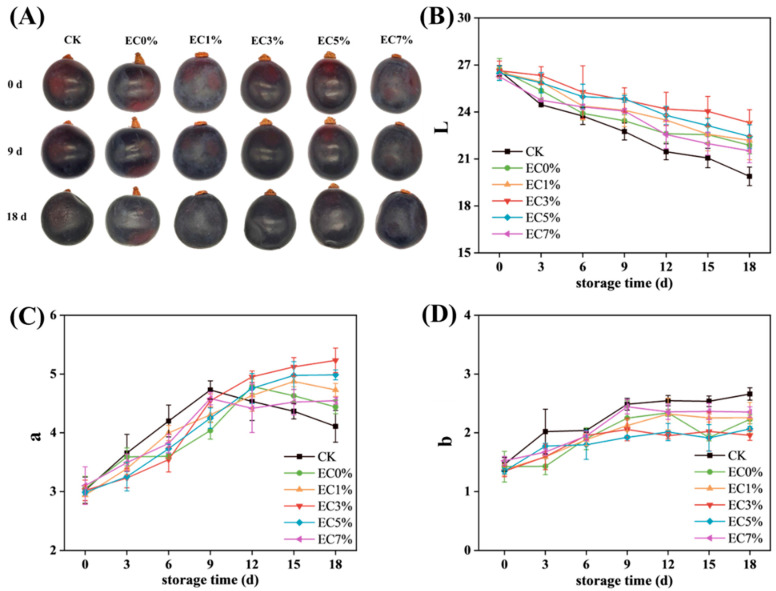
Appearance (**A**) and color difference (L (**B**), a (**C**), b (**D**)) of grapes treated with CMCS films containing 0%, 1.0% and 3.0%, 5.0%, 7.0% of citrus essential oil emulsion during storage.

As shown in Figure 6A, the effects of CMCS film coating versus direct air exposure on grape appearance were compared. At the end of storage, significant concavity and surface wrinkling were observed in some groups, with almost complete loss of gloss and substantial size reduction. In contrast, grapes from the EC1%, EC3%, and EC5% groups exhibited superior retention of morphological integrity and surface gloss. Changes in color during storage were analyzed using a chroma meter (L, a, b values)**,** since visual observation lacks precision. As shown in Figure 6B–D, the gradual decrease in L values indicated progressive surface darkening and gloss reduction, which may be attributed to enzymatic/non-enzymatic browning reactions and degradation of the surface wax layer [63]. The control group (CK) exhibited a significant L value reduction from 26.33 ± 0.33 to 19.89 ± 0.59 by 3 d, whereas EC5% and EC3% groups showed minimal changes during the initial 3 d, ultimately demonstrating the least L value decline at the end of storage (Figure 6B). Concurrently, the a values maintained positive values with an overall upward trend, potentially linked to anthocyanin accumulation during storage, which is a key determinant of red hue intensification in grapes [64]. Notably, the CK group exhibited an initial increase followed by a decrease in a values, with a faster ascending rate compared to treated groups (Figure 6C). This pattern may be attributed to accelerated anthocyanin accumulation during the early storage phase in the CK group, followed by senescence-driven anthocyanin degradation and oxidation, ultimately reducing a values. Although b values showed limited variation during storage, all groups demonstrated slight increases, with the CK group showing the most pronounced increment (Figure 6D). This phenomenon may result from the participation of anthocyanin degradation products in browning reactions [65]. Consistent with our findings, previous studies have documented analogous postharvest browning-induced chromatic alterations in grapes [66]. At the end of storage, all experimental groups exhibited quality deterioration in both morphological integrity and chromatic attributes. Notably, while EC0% demonstrated superior shape retention in grapes compared to EC7%, its color preservation efficacy was inferior to the EC1%–EC7% groups. This divergence is likely attributed to the lower WVP of EC0% relative to EC7%, combined with the enhanced antioxidant activity in EC7% formulations, which effectively inhibited anthocyanin oxidation degradation. The EC3% and EC5% groups demonstrated minimal alterations, indicating superior efficacy of emulsion-modified films in maintaining grape appearance and color stability.

#### 3.2.2. Changes in Postharvest Quality of Grapes

**Figure 7:**
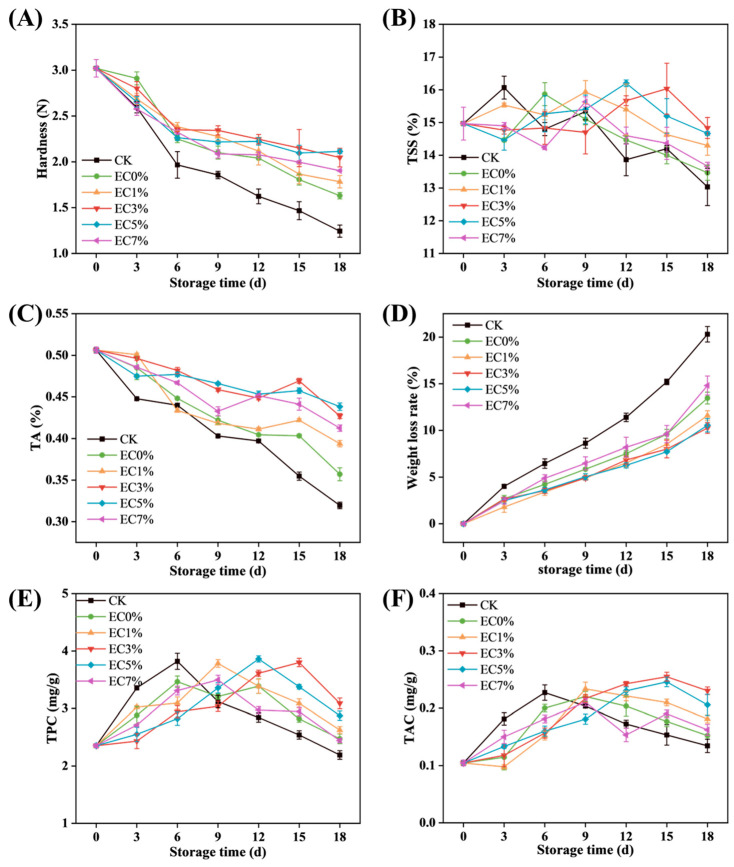
Changes in weight loss rate (**A**), hardness (**B**), TA (**C**), TSS (**D**), TPC (**E**), and TAC (**F**) of grapes treated with CMCS films containing 0%, 1.0% and 3.0%, 5.0%, 7.0% of citrus essential oil emulsion during storage.

Weight loss rate serves as a critical indicator for evaluating moisture depletion and quality degradation in postharvest fruits. In grapes, water loss induces epidermal wrinkling, gloss reduction, and spoilage [67]. As depicted in Figure 7A, progressive weight loss occurred across all groups during storage, attributable to water evaporation and organic matter depletion accelerated by respiratory metabolism [68]. At 18 d, CK exhibited the highest weight loss (20.31 ± 0.83%), while EC5% and EC3% groups demonstrated minimal losses of 10.26 ± 0.58% and 9.89 ± 1.08%, respectively, with no significant inter-group difference (*p* > 0.05). This correlation aligns with the WVP of films, confirming that film coatings effectively restrict evaporative pathways.

Hardness, determined by cellular structural integrity, serves as a critical indicator of fruit freshness, maturation stage, and senescence progression. As shown in Figure 7B, grape firmness exhibited a temporal decline during storage. This phenomenon is attributable to the combined effects of water/nutrient depletion and enzymatic degradation of cell wall components (e.g., pectin, cellulose, hemicellulose), leading to intercellular space expansion and tissue maceration [69,70]. The initial firmness of 3.02 ± 0.10 N decreased significantly to 1.24 ± 0.07 N (45.22% reduction) in the CK group after 18 d. All film-coated groups outperformed the control, with EC0% maintaining 1.63 ± 0.04 N, while EC3% and EC5% achieved superior firmness retention (2.05 ± 0.10 N and 2.11 ± 0.02 N, respectively). These results demonstrated that CEO emulsion additives assisted in the inhibition of cell wall polysaccharide degradation, thereby decelerating textural deterioration.

Titratable acidity (TA) significantly influences the properties and content of organic compounds, as well as the flavor, color, and stability of grapes. As shown in Figure 7C, TA levels in all groups decreased progressively during storage, likely due to the consumption of organic acids as substrates in respiratory metabolism. The CK group exhibited a rapid TA decline of 0.58 units after 3 d of storage, while film-coated groups maintained higher TA values with slower degradation rates. Among these, EC5% showed the best preservation effect, followed by EC3%. Total soluble solids (TSS) displayed an initial increase followed by a gradual decline (Figure 7D). During early storage, strong respiratory activity promoted the hydrolysis of macromolecular carbohydrates into soluble sugars, leading to TSS accumulation. Subsequently, reduced respiratory rates slowed hydrolysis, and soluble sugars were consumed as substrates, resulting in TSS decline [71,72]. The CK group showed a sharp TSS peak of 16.07% ± 0.35% at 3 d, followed by rapid depletion. Film coatings delayed the TSS peak occurrence (EC5%: 12 d; EC3%: 15 d), and coated groups maintained higher TSS values than CK at the end of storage. LEMES [73] observed similar trends in TA and TSS changes in gelatin-coated grapes, confirming reduced respiratory metabolism in treated samples. These results demonstrated that CEO essential oil-modified CMCS films enhanced barrier properties by effectively blocking oxygen ingress and moisture loss, thereby suppressing fruit respiration and metabolic activity.

#### 3.2.3. Changes in TPC and TAC of Grapes

The changes in TPC and TAC of grapes during storage are shown in Figure 7E,F. All groups exhibited an initial increase followed by a gradual decline in these compounds. This trend may arise from postharvest maturation processes, where phenolic substances accumulate during early storage, while subsequent senescence under storage conditions slows synthesis and accelerates degradation through oxidation [74]. Similar patterns were reported by Wu [75] in grape preservation studies. In the CK group, TPC increased rapidly from an initial value of 2.35 ± 0.03 mg·g^−1^ to a peak of 3.82 ± 0.03 mg·g^−1^ at 6 d, then declined sharply to 2.19 ± 0.08 mg·g^−1^ by the end of storage (Figure 7E). Film-coated groups delayed the peak occurrence of both TPC and TAC. The delayed peaks may result from the combined effects of the film’s barrier properties in reducing metabolic activity, the antioxidant capacity of essential oils in the emulsion, and the extended balance between synthesis and degradation phases. The EC3% and EC5% groups showed comparable efficacy in TPC and TAC retention. Consistent with the variation patterns observed for other nutritional components in preceding analyses, the EC0% group (without emulsion incorporation) exhibited inferior performance. This phenomenon may be attributed to the enhanced moisture and oxygen barrier properties provided by EC1% and EC3% emulsion-modified films, coupled with the elevated concentration of stabilized essential oil components in EC5% films, which conferred stronger antioxidant protection. In contrast, excessive emulsion incorporation in EC7% induced structural instability, leading to suboptimal preservation of TPC and TAC compared to films with lower emulsion concentrations.

#### 3.2.4. Enzyme Activities of Anthocyanin Synthesis

**Figure 8:**
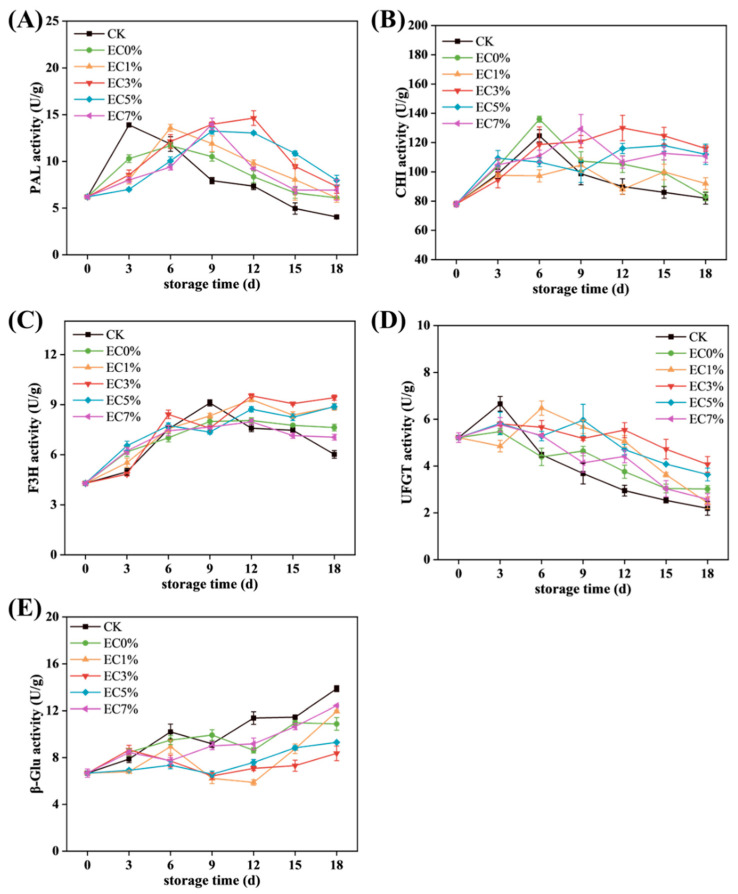
Changes in enzyme activity associated with anthocyanin metabolism (PAL activity (**A**), CHI activity (**B**), F3H activity (**C**), UFGT activity (**D**), β-GC activity (**E**)) of grapes treated with CMCS films containing 0%, 1.0% and 3.0%, 5.0%, 7.0% of citrus essential oil emulsion during storage.

Further analysis was conducted on anthocyanin metabolism-related enzyme activities. PAL, a key enzyme in the phenylpropanoid pathway, initiates the biosynthesis of anthocyanin precursors [76,77]. As shown in Figure 8A, PAL activity in the CK group surged rapidly during early storage due to water loss-induced stress, driving accelerated phenolic synthesis before declining sharply. Treated groups exhibited slightly lower initial PAL activity than CK, with delayed peak occurrence and higher residual activity at the end of storage, consistent with total phenolics and anthocyanin trends. CHI, F3H, and UFGT play critical roles in anthocyanin biosynthesis. CHI catalyzes chalcone cyclization, a prerequisite for flavonoid synthesis [6]. F3H hydroxylates flavanones to dihydroflavonols, direct precursors of anthocyanidin aglycones, with hydroxylation patterns determining anthocyanin subtypes [78,79]. CHI activity followed a rise–fall trajectory, while F3H in CK declined markedly post-mid-storage (after 9 d) (Figure 8B,C). F3H peaks lagged behind PAL and C3H, likely due to downstream substrate accumulation delays [80]. UFGT, the terminal rate-limiting enzyme, stabilizes anthocyanins via glycosylation. CK exhibited rapid UFGT peaking (attributed to upstream substrate abundance), whereas treated groups showed gradual increases or plateauing, with slower post-peak declines. Notably, EC3% maintained stable UFGT activity despite lower peak values (Figure 8D). β-GC hydrolyzes anthocyanin glycosidic bonds, accelerating degradation [8]. Its activity inversely correlated with anthocyanin content, rising throughout storage. CK displayed the highest β-GC activity, while EC3% retained the lowest levels (Figure 8E). The underlying mechanism for these enzymatic activity changes may involve water loss and essential oil release activating specific transcription factors (TFs) that regulate enzyme-coding genes [81]. MYB-TFs have been confirmed to bind and transcriptionally control key anthocyanin metabolic enzymes. For instance, burdock fructooligosaccharides downregulate *UFGT* expression by upregulating *MYBA2*, thereby limiting anthocyanin synthesis in postharvest grapes [82]. Concurrently, light exposure with appropriate temperatures (20 °C) synergistically enhances anthocyanin accumulation in harvested grape berries through elevated expression of *VlMYBA1-2* and *VlMYBA1-3*, which activate *F3H* and *UFGT* transcription [83]. Collectively, emulsion-modified films prolonged anthocyanin synthesis enzyme activity (PAL, CHI, F3H, UFGT) while suppressing β-GC activation, achieving delayed anthocyanin peaking and enhanced retention.

## 4. Conclusions

This study successfully developed CMCS films with enhanced water resistance, mechanical properties, antioxidant activity, and antimicrobial efficacy through CEO emulsion incorporation. The CEO emulsion-loaded CMCS films were able to reduce the postharvest nutrient reduction in grapes, effectively delaying the deterioration of the fruit and maintaining the quality. Meanwhile, the CEO emulsion-loaded CMCS films effectively maintained the color of the grapes by regulating the activity of enzymes related to the synthesis and degradation of anthocyanin, in which the EC3% and EC5% retained a better luster and appearance of grapes even after 18 d of storage. But this study has certain limitations: the absence of sensory analysis in grape quality evaluation and insufficient antimicrobial analysis against common grape pathogens. Future studies should focus on addressing these gaps, exploring the molecular mechanisms underlying preservation effects, and extending the film application to other perishable fruits and vegetables.

## Figures and Tables

**Table 1:** Color parameters of control and CMCS films containing 0%, 1.0% and 3.0%, 5.0%, 7.0% of citrus essential oil emulsion.

	L	a	b	ΔE
EC0%	95.09 ± 0.21 ^a^	−0.17 ± 0.08 ^a^	4.55 ± 0.25 ^d^	2.87 ± 0.29 ^d^
EC1%	94.79 ± 0.26 ^a^	−0.18 ± 0.03 ^a^	4.81 ± 0.25 ^cd^	3.22 ± 0.23 ^c^
EC3%	94.48 ± 0.15 ^b^	−0.19 ± 0.05 ^a^	4.81 ± 0.12 ^c^	3.35 ± 0.16 ^c^
EC5%	93.79 ± 0.54 ^c^	−0.19 ± 0.03 ^a^	5.32 ± 0.27 ^b^	4.14 ± 0.48 ^b^
EC7%	92.46 ± 0.22 ^d^	−0.28 ± 0.07 ^b^	7.56 ± 0.22 ^a^	6.73 ± 0.20 ^a^

Different letters in the same column represent significant differences (*p* < 0.05).

## Data Availability

The original contributions presented in the study are included in the article, further inquiries can be directed to the corresponding authors.
